# The conceptual framework and assessment methodology for the systematic reviews of community-based interventions for the prevention and control of infectious diseases of poverty

**DOI:** 10.1186/2049-9957-3-22

**Published:** 2014-07-31

**Authors:** Zohra S Lassi, Rehana A Salam, Jai K Das, Zulfiqar A Bhutta

**Affiliations:** 1 Division of Women and Child Health, The Aga Khan University, Karachi, Pakistan; 2 Center of Excellence in Women & Child Health, The Aga Khan University, Karachi, Pakistan; 3 Center for Global Child Health Hospital for Sick Children, Toronto, Canada

**Keywords:** IDoPs, Conceptual framework, Prevention and control, Community platforms, CHWs

## Abstract

This paper describes the conceptual framework and the methodology used to guide the systematic reviews of community-based interventions (CBIs) for the prevention and control of infectious diseases of poverty (IDoP). We adapted the conceptual framework from the 3ie work on the ‘Community-Based Intervention Packages for Preventing Maternal Morbidity and Mortality and Improving Neonatal Outcomes’ to aid in the analyzing of the existing CBIs for IDoP. The conceptual framework revolves around objectives, inputs, processes, outputs, outcomes, and impacts showing the theoretical linkages between the delivery of the interventions targeting these diseases through various community delivery platforms and the consequent health impacts. We also describe the methodology undertaken to conduct the systematic reviews and the meta-analyses.

## Multilingual abstracts

Please see Additional file 1 for translations of the abstract into the six official working languages of the United Nations.

## Introduction

**Figure 1:**
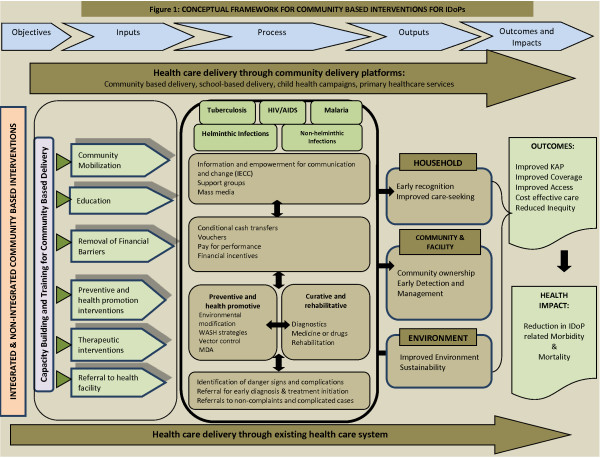
The conceptual framework for integrated community-based interventions for NTDs.

Infectious diseases of poverty (IDoPs), including neglected tropical diseases (NTDs), malaria, tuberculosis (TB) and HIV/AIDS, disproportionately affect the poorest populations in the world. A large proportion of infectious diseases in low- and middle- income countries (LMICs) are entirely avoidable or treatable with existing interventions and drugs [1]. The previous publication has discussed in detail the prevailing burden, distribution, and existing interventions for the prevention and control of IDoPs, while this paper describes the conceptual framework and methods used to guide the systematic reviews. We adapted the conceptual framework from the 3ie work [2] to analyze how existing community-based interventions (CBIs) can prevent and control IDoPs (see Figure 1). The 3ie framework was used to evaluate the effectiveness of CBI packages for maternal, perinatal, and neonatal health outcomes. It shows the theoretical linkages between the CBI packages, utilizing community health workers (CHWs), as well as the health outcomes and access to care [2]. We have modified this framework to incorporate the CBIs pertaining to IDoPs, and their impacts.

## Review

### Conceptual framework

The conceptual framework revolves around objectives, inputs, processes, outputs, outcomes, and impacts. It shows linkages between the delivery of the interventions targeting IDoPs through various community delivery platforms and their consequent impacts. The framework is based on capacity building and training for the delivery of preventive and curative interventions in community settings, either integrated with the existing health services or vertically administered. Some of these CBIs are comprehensive and target a range of diseases. Such CBIs include community mobilization, education and training, financial incentives, and referrals to health facilities. Other CBIs, however, are disease focused and include, for instance, the provision of disease specific preventive and curative chemotherapy. Community mobilization comprises the formation of support groups, educating community members on recognizing the danger signs early, and the use of mass media campaigns. The removal of financial barriers involves conditional cash transfers (CCTs), vouchers, and pay for performance. Community-based health promotion and preventive interventions include environmental modification; water, sanitation, and hygiene (WASH) interventions; vector control; and mass drug administration (MDA). Curative interventions include disease-specific diagnosis, treatment, and rehabilitation. Referral encompasses early diagnosis, treatment initiation, and institutional care for complicated and non-complicated cases. These interventions, when implemented in a synergistic manner, will lead to positive changes at the household, community, and facility levels. These changes would in turn improve knowledge, attitude and practices (KAP), coverage and access, and lead to reduced morbidity and mortality related to IDoPs. In this paper, we describe the components of our conceptual framework on all levels: inputs, processes, outputs, outcomes, and impacts. This paper also details the methodology used for conducting the systematic reviews and meta-analyses.

### Inputs and processes

#### ***Capacity building and training***

According to the World Health Organization (WHO), there is a shortage of 7.2 million healthcare workers globally, with developing countries facing the heft of this burden [3]. Fifty-seven countries have critical shortages of healthcare workers and 36 of these are in Sub-Saharan Africa, which has 25% of the world’s burden of disease with only 3% of the world’s healthcare workers to cater to it. Health service delivery through an alternate cadre of skilled and semi-skilled workers has been practiced all over the world for the past several decades, however more recently, due to the growing human resource crisis especially in LMICs, the role of task shifting has re-emerged in order to provide services to ‘hard-to-reach’ groups [4-7]. Thus, increasing the number of CHWs, training and educating them, providing them with incentives, and improving the infrastructure to reduce turnover is crucial for the community-based prevention and control of these infectious diseases. Community health worker programs have increasingly been receiving greater attention over the last few years and a number of publications have documented the impact of such programs [8-11]. The Access Project which sets out to control NTDs in Rwanda and the lymphatic filariasis (LF) control program in Togo are among a few of the recent examples where CHWs were successfully employed and trained to undertake screening and MDA campaigns to achieve effective control [12,13]. Training school teachers and personnel is another cost-effective way to administer MDA and successful examples exist where teachers have administered albendazole (ABZ) and praziquantel (PZQ) for soil-transmitted helminthiasis (STH) and schistosomiasis. The Bangladesh Rural Advancement Committee (BRAC) is also one of the laudable initiatives, under which 70,000 CHWs continue to work and are connected to a functioning health system [14]. These CHWs earn an income by identifying TB patients and ensuring treatment completion by providing directly observed treatment. An alternative cadre of CHWs involved in drug and equipment supply are the community-based vendors and medicine sellers. Medicine sellers have played an important role in facilitating access to essential medicines and the distribution of insecticide-treated nets (ITNs) in Sub-Saharan Africa. The training and capacity building of these individuals has aided in bringing about appropriate demand, and in enhancing quality assurance and community acceptability [14].

#### ***Community mobilization and education***

It is well recognized that community participation is important for the successful delivery of health services at the community level [15]. Community-based support groups and women’s groups comprising community representatives are increasingly becoming a core component of community service packages. It is a key part of successful drug compliance for preventive as well as therapeutic regimens as it emphasizes the importance of treatment completion, helps to communicate key concepts, ensures sustainability and accountability, and addresses the myths related to IDoPs. Health education plays a pivotal role in prevention and control as many of these diseases can be successfully prevented and controlled with vector control and WASH strategies. Many NTD programs include community mobilization and education as core components, for example, the Tanzania NTD Control Program supported by Envision and the End NTDs in Africa programs in Ghana and Sierra Leone.

#### ***Removal of financial barriers***

In resource-limited settings, IDoPs are compounded by low investment in health, lack of comprehensive health financing policies, limited financial access to health, and extensive out-of-pocket payments. To ameliorate poverty and improve healthcare access for the poor and marginalized population, various financial mechanisms have been devised, tested, and implemented at scale. These strategies improve the uptake of desired health services that are otherwise constrained by the lack of financial prowess, and also provide household economic stability and help alleviate poverty. They involve the provision of monetary benefits as a source of motivation for desired health-related actions and free access to basic healthcare, thus creating a demand for health services. Diverse and innovative financial support platforms are being implemented in some of the fragile states such as Cambodia, Afghanistan, Pakistan and Haiti, as well as in the more established economies of Latin American countries, to improve overall maternal and child health [16,17]. In the domain of IDoPs, potential promising impacts have been seen for improving the uptake of ITNs through vouchers and social marketing, while cash transfers and food and nutrition support have shown some impact on reducing vulnerability to HIV/AIDS among adolescent girls and young women [18-20]. However, financial incentives are yet to be formally evaluated for effectiveness against IDoP and any measurable effects from rigorous evaluations have not been reported as yet [18-20].

#### ***Health promotion and preventive interventions***

To break the cycle of infections, health promotion and preventive interventions are critical. Since many of these diseases are vector borne or transmitted through poor sanitation and hygiene conditions, they can be averted if effective preventive measures are in place. For effective control of dengue, chagas, human African trypanosomiasis, leishmaniasis, dracunculiasis, LF, onchocerciasis and schistosomiasis, vector control mainly relies on insecticides. An effective sanitation infrastructure is ideal for interrupting the transmission of many diseases, however, the resources are limited and not sustainable in developing countries [21]. Preventive chemotherapy is the most feasible and cost-effective measure as safe and effective drugs exist [22,23]. Programs to control NTDs predominantly employ MDA to treat high-risk population [24] and WHO endorses this strategy as ‘preventative chemotherapy’ [25]. For some of these diseases, chemotherapy is common and can be administered concurrently, for example, ABZ is given to treat LF and STH, while ivermectin (IVR) can be given for both LF and onchocerciasis [26]. Other preventive strategies involve community-based vector control measures such as insecticide spraying and ITNs for dengue, chagas, and leishmaniasis.

Of the various measures in place for malaria prevention, key interventions include indoor residual spraying (IRS), use of ITNs, intermittent preventive therapy (IPT), presumptive treatment, and mass awareness. In Sub-Saharan Africa, the wide-scale implementation of ITNs is now one of the main strategies to reduce malaria morbidity and mortality [27]. The ITNs have the potential to hugely affect the spread of this disease, and their use has been associated with reduced mortality among children [28-30]. The WHO also recommends IPT for pregnant women and infants in highly endemic areas.

For HIV prevention, interventions must involve behavioral changes to reduce HIV risk and promote awareness about condom use, safe sex practices, voluntary testing and counseling, and voluntary male circumcision. Couples counseling and condom distribution have proven effective in many countries such as Kenya and Zambia, however, many countries still lack a comprehensive strategy for rolling out these programmatic approaches [19].

#### ***Therapeutic interventions***

Therapeutic control of NTDs recommended by the WHO involves periodic administration of ABZ and mebendazole (MBZ) for STH, PZQ for schistosomiasis, and IVR or diethylcarbamazine (DEC) for LF once or twice a year depending on the baseline prevalence among the populations at risk [31]. For trachoma, the WHO recommends the SAFE (surgery, azithromycin, facial cleanliness, and environmental hygiene) strategy, while for leprosy, multidrug therapy is recommended. For the treatment of malaria, the artemisinin-based combination therapy is recommended. Tuberculosis is completely curable through WHO’s Stop TB Strategy (founded on the core of the DOTS strategy). It is based on the prompt diagnosis of the active disease and followed by supervised, short-course combination chemotherapy, as recommended. Ensuring completion of treatment is crucial for the prevention of relapse and secondary drug resistance. Since 1995, 41 million people have been successfully treated and up to six million lives have been saved through DOTS and the Stop TB Strategy [32]. The treatment of HIV involves antiretroviral therapy (ART) regimens to effectively reduce the risk of HIV transmission. Pregnant women living with HIV should also be treated with recommended regimens to prevent mother-to-child transmission (MTCT) [19].

#### ***Referrals***

An effective referral system ensures a close relationship between all levels of the health system and helps to ensure the best possible health care [33]. Support for CHWs by experienced staff from the district health facility helps build capacity and enhance access to better quality care. Programs focusing on the prevention and management of IDoPs should also strengthen referral services alongside active case detection to manage and rehabilitate diagnosed chronic cases with incapacitating conditions. This will ensure optimal care at appropriate time, avoid unnecessary costs, and provide timely treatment.

### Community delivery platforms

Community delivery platforms deliver a range of health services to meet local community needs and are increasingly being advocated to improve nutrition and control diseases [8,9]. Various community delivery platforms can be utilized for the prevention and control of IDoPs. The school system offers an ideal setting for deworming and provision of health education messages to children. Anthelmintics can be delivered by school teachers as medicines are safe to administer with minimal training, thus making the practice cost effective. In 2010, a review of costs in seven countries in four WHO regions estimated that the average cost of treating one million children was US$ 72,000 (or 7.2 cents per child). This estimate included procurement and distribution of medicines, training of teachers, and supervision and monitoring [34]. Vaccination and supplementary campaigns (for example, vitamin A distribution) also provide opportunities to deworm preschool-age children, and it is shown that deworming usually increases the coverage of vaccination and supplementary campaigns [35]. Since the infrastructure and personnel are already in place to distribute vitamins or vaccines, they can easily administer deworming tablets at a minimum added cost. Pregnant women and women of reproductive age can be easily targeted for IPT and ART through existing community-based maternal and child health services and this is feasible even in resource-poor settings [36]. Large-scale administration of these drugs should be incorporated, along with other associated activities such as staff training, data collection, and development of materials for advocacy and community mobilization.

The interventions identified in our conceptual framework are diverse, but the ultimate goal of these interventions is to reduce the prevalence and morbidities associated with IDoPs. We would like to emphasize that these components work in parallel with each other to bring about a synergistic effect. With this understanding of the effective model of delivering CBIs for prevention and control of IDoPs, we aim to achieve improvements at household (knowledge, behavior, and care seeking); community/facility (ownership, quality of care, early detection, and management); and environmental (sustainability and improved environment) levels. These outputs would eventually lead to broader outcomes and impacts such as improved access and coverage of interventions and consequent decreases in prevalence, associated morbidities, and mortalities of these diseases.

## Methods

We conducted systematic reviews of all studies focusing on existing community-based interventions for IDoPs. Guided by our conceptual framework, we focused on studies evaluating the effectiveness of CBIs targeting the 14 major NTDs (leishmaniasis, human African trypanosomiasis, chagas disease, dengue, trachoma, leprosy, buruli ulcer, and the helminthes including hookworm, ascariasis, trichuriasis, LF, onchocerciasis, dracunculiasis and schistosomiasis); malaria; TB; and HIV/AIDs compared to routine healthcare delivery. For these reviews, we have categorized NTDs into helminthic and non-helminthic diseases, and report the findings accordingly in separate papers. Helminthic diseases included STH such as ascariasis, hookworm and trichuriasis, along with schistosomiasis, LF, onchocerciasis, and dracunculiasis. Non-helminthic diseases included dengue, African trypanosomiasis, chagas, leishmaniasis, trachoma, leprosy, and buruli ulcer. In this review, we have used the following definitions for CBIs, integrated CBIs, and additional training:

• A ‘community-based intervention’ was defined as any intervention or care package delivered by healthcare personnel or lay individuals at home, village, or any defined community setting, but not in a health facility. Such intervention packages might include additional training for outreach workers, namely lady health workers/visitors, community midwives, community/village health workers, or facilitators to deliver interventions related to prevention and control of the outlined infectious diseases. The CBIs also included any financial interventions to improve uptake of the desired health services which are otherwise constrained by lack of financial resources.

• ‘Integrated CBIs’ were defined as interventions merged into any existing program, for example, routine maternal child health programs or primary healthcare setups.

• ‘Additional training’ was defined as any training other than the routine training that healthcare workers or CHWs received from governmental or non-governmental organizations (NGOs), and could include didactic sessions, lectures, and supervised hands-on training in a healthcare facility and/or within the community.

We considered all available existing randomized, quasi-randomized, and before-and-after studies measuring the impact of CBIs to prevent and treat IDoPs. In addition, other less rigorous study designs such as observational (cohort and case–control) and descriptive studies were also reviewed to understand the context within which they were implemented, typology of healthcare providers, types of intervention delivered, and reported results.

### Search strategy and selection criteria

Studies were included if intervention was delivered within community setting and if reported outcomes were relevant to the diseases under review. We systematically reviewed literature published before May 2013 to identify relevant studies. Searches were conducted in PubMed, Cochrane Libraries, Embase, and WHO Regional Databases to identify all published and unpublished studies. Additional studies were identified by hand searching references from the included studies. A broad search strategy was used that included a combination of appropriate keywords, medical subject headings (MeSH), and free text terms i.e. [(“Infectious diseases” OR “infectious diseases in poor*” OR “infectious diseases of poverty” OR “malaria” OR “tuberculosis” OR “TB” OR “HIV/AIDS” OR “neglected tropical disease*” OR “NTD” OR “leishmaniasis” OR “human African trypanosomiasis” OR “chagas disease” OR “dengue” OR “trachoma” OR “leprosy” OR “buruli ulcer” OR “helminth infection*” OR “STH” OR “soil-transmitted helminth*” OR “ascariasis” OR “trichuriasis” OR “lymphatic filariasis” OR “onchocerciasis” OR “dracunculiasis” OR “schistosomiasis”) AND (“community” OR “community health aides” OR “primary health care” OR “community health worker*” OR “lay health worker*” OR “mid-level health worker*” OR “community-based interventions” OR “outreach”)].

The abstracts and the full sources were screened by two of the authors to identify if the studies adhered to the set inclusion criteria. Any argument on selecting studies between these two authors was resolved by the third author. After full text retrieval of all eligible studies, double data was abstracted from each study into a standardized form to elicit the following information:

• Study design;

• Country, including settings (urban/rural);

• Intervention type (preventive/therapeutic);

• Intervention description;

• Mode of delivery (integrated or non-integrated); and

• Outcomes assessed.

### Quality assessment

Two review authors independently assessed the risk of bias for each study using the Cochrane risk of bias assessment criteria [37]. This was based on sequence generation (checking for possible selection bias); allocation concealment (checking for possible selection bias); blinding (checking for possible performance bias); incomplete outcome data (checking for possible attrition bias through withdrawals, dropouts, protocol deviations); selective reporting bias; or any other sources of bias. The above mentioned criteria were rated as ‘adequately done’, ‘not done’, or ‘unclear’. The level of attrition was noted for each study and its impact on the overall assessment of treatment effect was explored by using sensitivity analysis, where possible. For all outcomes, we carried out the analysis, as far as possible, on an intention-to-treat basis.

### Statistical analysis

We conducted a meta-analysis for individual studies and pooled statistics were reported as the relative risk (RR) for categorical variables and standard mean difference (SMD) for continuous variables between the experimental and control groups with 95% confidence intervals (CIs). Mantel-Haenszel pooled RR and corresponding 95% CIs were reported when there was no evidence of heterogeneity. The DerSimonian and Laird pooled RR and corresponding 95% CIs were reported where there was an unexplained heterogeneity. All analyses were conducted using the software Review Manager 5.1.

### Assessment of heterogeneity

Heterogeneity was quantified by Chi^2^ and I^2^, which can be interpreted as the percentage of the total variation between studies that is attributable to heterogeneity rather than to chance, a low p-value (less than 0.1), or a large chi-squared statistic relative to its degree of freedom. I^2^ values greater than 50% were taken as a substantial and high heterogeneity. In situations of high heterogeneity, causes were explored by sensitivity analysis and random effect models were used. The primary comparison was for the community delivered interventions versus routine health service delivery. However, where possible, we also attempted to perform a subgroup analysis for the integrated versus the non-integrated delivery. The subgroup analysis was done for the following comparisons:

• A community-based delivery versus a routine delivery of interventions;

• Integrated CBIs versus non-integrated CBIs;

• Evidence from randomized and quasi-randomized studies versus pre-post study designs;

• A school-based delivery versus a routine delivery; and

• Preventive CBIs versus therapeutic CBIs.

• Types of intervention.

Sensitivity analysis was carried out to explain possible heterogeneity in the summary estimates. We conducted sensitivity analysis by removing high risk of bias study from the pooled estimate and compared the estimate with and without the study data. The level of attrition was noted for each study and its impact on the overall assessment of the effect of treatment was explored by using sensitivity analysis.

### Qualitative synthesis

We also attempted to qualitatively synthesize the findings reported in the included studies for other pragmatic parameters highlighted in our conceptual framework that could not be quantitatively meta-analyzed. These factors included intervention coverage, challenges/barriers, enabling factors, aspects related to integrated delivery, monitoring and evaluations, equity, etc. Given this causal model, we aimed to systematically analyze available evidence on the effectiveness of CBIs to prevent and treat IDoPs, including helminthic NTDs, non-helminthic NTDs, malaria, HIV/AIDs, and TB.

## Conclusion

Based on the conceptual framework and the described methodology, we have evaluated the impact of CBIs on the outlined health outcomes. The findings from the systematic reviews on helminthic NTDs non-helminthic NTDs, malaria, HIV/AIDS, and TB are reported in separate papers.

## Abbreviations

ART: Antiretroviral therapy; CBI: Community-based intervention; CHW: Community health worker; CI: Confidence interval; HIV/AIDS: Human immunodeficiency virus/acquired immunodeficiency syndrome; IDoP: Infectious disease of poverty; IPT: Intermittent preventive therapy; IRS: Indoor residual spraying; ITN: Insecticide-treated net; KAP: Knowledge, attitude, and practice; LMIC: Low- middle-income country; MDA: Mass drug administration; MTCT: Mother-to-child transmission; NTD: Neglected tropical disease; RR: Relative risk; SAFE: Surgery, azithromycin, facial cleanliness, and environmental hygiene; SMD: Standard mean difference; STH: Soil-transmitted helminthiasis; TB: Tuberculosis; WASH: Water, sanitation, and hygiene; WHO: World Health Organization.

## Competing interests

The authors declare that they have no financial or non-financial competing interests.

## Authors’ contributions

ZAB was responsible for designing and coordinating the review. All authors contributed, read and approved the final manuscript.

## Supplementary Material

Additional file 1 Multilingual abstracts in the six official working languages of the United Nations. Click here for file
