# Supplementary material for: The conceptual framework and assessment methodology for the systematic reviews of community-based interventions for the prevention and control of infectious diseases of poverty
Source: Infect Dis Poverty. 2014 Jul 31;3:22. doi: 10.1186/2049-9957-3-22 (PMC4124965; doi:10.1186/2049-9957-3-22)
Supplement: Additional file 1 — Multilingual abstracts in the six official working languages of the United Nations. [file 2049-9957-3-22-S1.pdf]

Translation of the abstract into the six official working languages of the United Nations

إطار العمل المفاهيمي ومنهجية التقييم للمراجعة الممنهجة للتدخلات المجتمعية من أجل مكافحة وإدارة الأمراض المعدية المرتبطة بالفقر (IDoPs).

زهرة س لاسي، ريحانة أ سلام، جاي ك داس، ذو الفقار أ بوتا

ملخص

تشرح هذه الورقة إطار العمل المفاهيمي والمنهجية المستخدمة كدليل المراجعة الممنهجة للتدخلات المجتمعية (CBIs) لمكافحة وإدارة الأمراض المعدية المرتبطة بالفقر (IDoPs). قمنا بتبني إطار العمل المفاهيمي من عمل 3ie في مجال "مجموعات التدخل المجتمعية لمكافحة الأمراض والوفيات الناتجة عن الولادة وتحسين نتائج حديثي الولادة" بهدف المساعدة في تحليل CBIs الحالية في حالات IDoPs. يتمحور إطار العمل المفاهيمي حول أهداف، مدخلات، عمليات، مخرجات، نتائج والآثار التي توضح الروابط النظرية بين تنفيذ التدخلات التي تستهدف هذه الأمراض عبر منصات توزيع مجتمعية والآثار الصحية الناتجة عنها. كما نشرح أيضا المنهجية المتبعة لإجراء المراجعات المنهجية وتحليل التحاليل الإحصائية.

Translated from English version into Arabic by Assem Mazloum, through

TRANSLATORS
WITHOUT BORDERS

贫困所致传染病防控的社区干预系统综述之基本概念与评价方法

Zohra S Lassi, Rehana A Salam, Jai K Das, Zulfiqar A Bhutta

摘要

本文描述了关于指导开展贫困所致传染病防控的社区干预系统综述的一些基本概念与方法。将“降低母体发病率和死亡率与改善新生儿结局的社区干预”项目的基本概念用于分析已有的贫困所致传染病防控的社区干预措施。基本概念围绕目的、投入、过程、产出、结局和影响来体现不同社区平台实施这些疾病的社区干预与后续健康影响之间的理论联系。本文还介绍了开展系统综述和 Meta 分析的一些方法。

Translated from English version into Chinese by Yin Jian-hai, through

CHINESE CENTER FOR DISEASE CONTROL AND PREVENTION
NATIONAL INSTITUTE FOR PARASITIC DISEASES

Cadre conceptuel et méthode d'évaluation pour la revue systématique des interventions communautaires visant la prévention et le contrôle des MILP

Zohra S Lassi, Rehana A Salam, Jai K Das, Zulfiqar A Bhutta

Résumé

Le présent article décrit le cadre conceptuel et la méthode d'évaluation employés pour la revue systématique des interventions communautaires visant la prévention et le contrôle des maladies infectieuses liées à la pauvreté (MILP). Nous avons emprunté au 3ie son cadre conceptuel de travail sur « les ensembles de mesures communautaires pour la prévention de la morbidité et de la morbidité maternelles et l'amélioration des résultats néonatals » et l'avons adapté à l'analyse des interventions existantes visant les MILP. Le cadre conceptuel est axé sur des objectifs, des intrants, des processus, des produits, des résultats et des impacts, faisant apparaître des liens théoriques entre la réalisation des interventions visant ces maladies par différentes plates-formes communautaires et les impacts sanitaires qui en découlent. Nous décrivons également la méthodologie employée pour conduire les revues systématiques et les méta-analyses.

Translated from English version into French by Suzanne Assenat, through

TRANSLATORS
WITHOUT BORDERS

Концептуальная основа и методология оценки для проведения систематических обзоров территориальных мероприятий по предупреждению и контролю инфекционных болезней бедности

Зохра С Ласси, Рейяна А Салам, Джай К Дас, Зульфикар А Бхутта

Реферат

В данной работе описывается концептуальная основа и методология, используемые для проведения систематических обзоров территориальных мероприятий (CBIs) по предупреждению и контролю инфекционных болезней бедности (IDoPs). Нами была адаптирована концептуальная основа проекта 3ie по «Система территориальных мероприятий по предупреждению материнской заболеваемости и смертности и улучшению неонатального развития» с целью помочь в анализе существующих территориальных мероприятий (CBIs) по предупреждению и контролю инфекционных болезней бедности (IDoPs). Концептуальная основа сосредотачивает внимание на целях, исходных данных, процессах, результатах и последствиях и показывает теоретические связи между постановкой целей таких мероприятий с помощью различных платформ, имеющихся в местных сообществах, и вытекающим из этого воздействием на здоровье. Мы описываем также методологию, использованную для проведения систематических обзоров и мета-анализа.

Translated from English version into Russian by Alena Hrybouskaya, through

TRANSLATORS
WITHOUT BORDERS

El marco de referencia conceptual metodología de evaluación para las revisiones sistemáticas de las intervenciones basadas en la comunidad para la prevención y el control de Enfermedades Infecciosas de la Pobreza.

Zohra S Lassi, Rehana A Salam, Jai K Das, Zulfiqar A Bhutta

Resumen

Este documento describe el marco de referencia conceptual metodología de evaluación para las revisiones sistemáticas de las intervenciones basadas en la comunidad para la prevención y el control de Enfermedades Infecciosas de la Pobreza (*IDoPs*, en inglés). Adaptamos el marco de referencia conceptual del trabajo 3ie acerca de “Paquetes de Intervención Basados en Comunidades para la Prevención de Morbilidad y Mortalidad Maternales y la Optimización de los Resultados en Recién Nacidos, a fin de ayudar en el análisis de las CBIs existentes de Enfermedades Infecciosas de la Pobreza (*IDoPs*, en inglés). El marco de referencia conceptual enmarca los objetivos, entradas, procesos, salidas, resultados e impactos que muestran las vinculaciones teóricas entre la entrega de intervenciones orientadas a estas enfermedades mediante diversas plataformas de entrega de la comunidad y los impactos subsiguientes en la salud. También describimos la metodología asumida para llevar a cabo las revisiones sistemáticas y los meta-análisis.

Translated from English version into Spanish by Nana Rodriguez, through

TRANSLATORS
WITHOUT BORDERS
